# Long-term clinical outcomes of modular endoprosthetic reconstruction for primary bone tumours of the proximal femur

**DOI:** 10.1016/j.jbo.2026.100783

**Published:** 2026-07-07

**Authors:** P.T.J. Sanders, S.F. van de Vusse, M.P.A. Bus, M. Fiocco, J.A.M. Bramer, G.R. Schaap, M.A.J. van de Sande, P.D.S. Dijkstra

**Affiliations:** a Leiden University Medical Center, Department of Orthopaedic Surgery, Leiden, the Netherlands; b Leiden University Medical Center, Department of Data Science, section Medical Statistics, Leiden, the Netherlands; c Leiden University, Mathematical Institute, Leiden, the Netherlands; d Princess Máxima Center for Paediatric Oncology, Utrecht, the Netherlands; e Academic Medical Center, Department of Orthopaedic Surgery, Amsterdam, the Netherlands

**Keywords:** Proximal femoral reconstruction, Endoprosthetic reconstruction, Bipolar hemiarthroplasty, Bone tumours, Limb salvage surgery, Long-term outcomes

## Abstract

Endoprosthetic reconstruction of the proximal femur is a well-accepted technique in the treatment of bone tumours. However, controversies remain regarding the optimal reconstructive technique. For instance, there is little evidence to support resurfacing of the acetabulum. Our preferred technique during recent years was hemiarthroplasty with a bipolar femoral head and an attachment tube for tendinous reinsertion. Aim of this study was to evaluate the long-term clinical outcomes of these reconstructions. All consecutive patients in whom a MUTARS® proximal femoral replacement was performed between 1999 and 2023 for a primary bone tumour were retrospectively evaluated, with a minimum follow-up of 24 months. Sixty-four patients matched inclusion criteria. Mean age was 52 years (8 to 89). Chondrosarcoma was the predominant diagnosis (*n* = 37, 58%), followed by osteosarcoma (*n* = 16, 25%). At review, 36 patients (56%) were alive. Median follow-up was 10.1 years (IQR 4.5–14.7). Fifty-four reconstructions (84%) were bipolar hemiarthroplasties. Twenty-eight (44%) implants were silver-coated. Median reconstruction length was 18 cm (8 to 45). Dislocation occurred in five bipolar hemiarthroplasties (9%) and in three total hip arthroplasties (33%). Aseptic loosening was not observed. One periprosthetic fracture was observed (2%). Deep infections occurred in eight reconstructions (13%). The cumulative incidence of mechanical failure at five, ten, and fifteen years was 11.8% (95% CI 3.4–20.3), 22.6% (95% CI 10.1–35.2), and 29.7% (95% CI 14.4–44.9). In our experience, bipolar hemiarthroplasty appears to be a viable technique for reconstruction of the proximal femur after bone tumour resection, with a low rate of mechanical failure and acetabular wear at long-term follow-up.

## Highlights

• Longest follow-up of MUTARS® proximal femoral replacement to date.
• Bipolar hemiarthroplasty showed low acetabular revision after long follow-up.
• Implant retained in 84%; limb salvage achieved in 94% of patients.

## 1 Introduction

Several techniques have been described for reconstruction of proximal femoral defects, including the use of allograft-prosthetic composites [1], [2], [3], [4] and custom-made [5] or modular [6], [7], [8] endoprostheses. Advantages of allografts are bone stock restoration and the possibility to re-attach soft tissues [4]. However, their use has been associated with high complication rate (40–60%), due to infection, fracture and non-union [3], [9]. Endoprostheses represent an attractive option because they allow for early weight bearing. Modular implants additionally offer the convenience of being available off-the-shelf and the possibility of intraoperative adjustment [6], [10], [11]. As a result, modular endoprostheses have become increasingly popular, not only in treatment of primary bone tumours but also of metastatic disease [6].

To date, most studies on proximal femoral endoprosthetic reconstruction report mainly on patients treated for bone metastases. As this subgroup has limited life expectancy, death as a competing event results in an underestimation of the true risk of complications among patients who survive. Studies focusing merely on the clinical outcomes of proximal femoral reconstruction for patients with substantially longer expected survival are lacking. In addition, numerous controversies persist related to the optimal reconstructive technique. For instance, there is little evidence to support resurfacing of the acetabulum. In this study, we aimed to assess complications, risk factors for complications, and cumulative incidences of implant failure to evaluate the durability of proximal femoral reconstruction with a MUTARS® modular endoprosthesis after primary tumour resection.

## 2 Patients and methods

All consecutive patients in whom a proximal femoral replacement after primary tumour resection was performed with a MUTARS® proximal femoral prosthesis (implantcast, Buxtehude, Germany) were retrospectively identified through the institutional databases. Patients were followed for a minimum of 2 years or until death; all procedures took place between 1999 and 2023. Fifty-three reconstructions (83%) were performed in centre 1, eleven (17%) in centre 2.

Sixty-four patients matched our inclusion criteria. Mean age at surgery was 52 years (8 to 89). Predominant diagnoses were chondrosarcoma (*n* = 37, 58%), osteosarcoma (*n* = 16, 25%) and Ewing sarcoma (*n* = 4, 6%) (Table 1). Fifteen (23%) had surgery around the proximal femur prior to the index procedure, including a variety of osteosyntheses and local treatments (Table 2). At review, 36 patients (56%) were alive. Twenty-eight patients (44%) died during follow-up. Median follow-up was calculated using the reverse Kaplan-Meier method and was equal to 10.1 years (Interquartile range (IQR) 4.5 to 14.7) [12].

**Table 1:** Study data.

	BHA (%)	THA (%)	Total (%)
Sex			
Male	31 (57)	7 (78)	38 (59)
American Society of Anaesthesiologists (ASA) score [40]			
ASA 1	8 (15)	4 (44)	12 (19)
ASA 2	38 (70)	4 (44)	43 (67)
ASA 3	5 (9)	1 (11)	6 (9)
Diagnosis			
Chondrosarcoma	30 (56)	6 (67)	37 (58)
Osteosarcoma	15 (28)	1 (11)	16 (25)
Ewing sarcoma	4 (7)	0 (0)	4 (6)
Other	5 (9)	2 (22)	7 (11)
Pathological fracture	11 (20)	2 (22)	13 (20)
Adjuvant therapies			
Neo-adjuvant chemotherapy	13 (24)	1 (11)	14 (22)
Adjuvant chemotherapy	17 (32)	0 (0)	17 (27)
Neo-adjuvant radiotherapy	1 (2)	0 (0)	1 (2)
Adjuvant radiotherapy	3 (6)	0 (0)	3 (5)
Reconstruction details			
Bipolar hemiarthroplasty	54 (100)	0 (0)	54 (84)
Total hip arthroplasty	0 (0)	9 (100)	9 (14)
Attachment tube	49 (91)	8 (89)	58 (91)
Uncemented	43 (80)	6 (67))	50 (78)
Hydroxyapatite-coated	29 (67⁎)	5 (83⁎)	34 (68⁎)
Silver coating	24 (44)	4 (44)	28 (44)
Median (range)			
Length of reconstruction (cm)	18 (8–45)	20 (12−32)	18 (8–45)
Surgical duration (min)	200 (111–540)	229 (190–384)	205 (111–540)
Blood loss (ml)	425 (35–4000)	2100 (500–3000)	500 (35–4000)
Postoperative hospital stay (days)	8 (3–51)	13 (5–16)	8 (3–51)

Abbreviations: BHA, bipolar hemiarthroplasty; THA, total hip arthroplasty.

⁎ Percentage of uncemented reconstructions.

**Table 2:** Previous surgery.

Procedure	Reconstruction	Number	Reasons for reconstruction failure
Curettage	Cancellous bone grafting	5	Tumour recurrence (n = 5)
Osteosynthesis	Dynamic hip screw	2	–
	Intramedullary nail	4	–
Arthroplasty	Total hip prosthesis	4	–

**Fig. 1:**
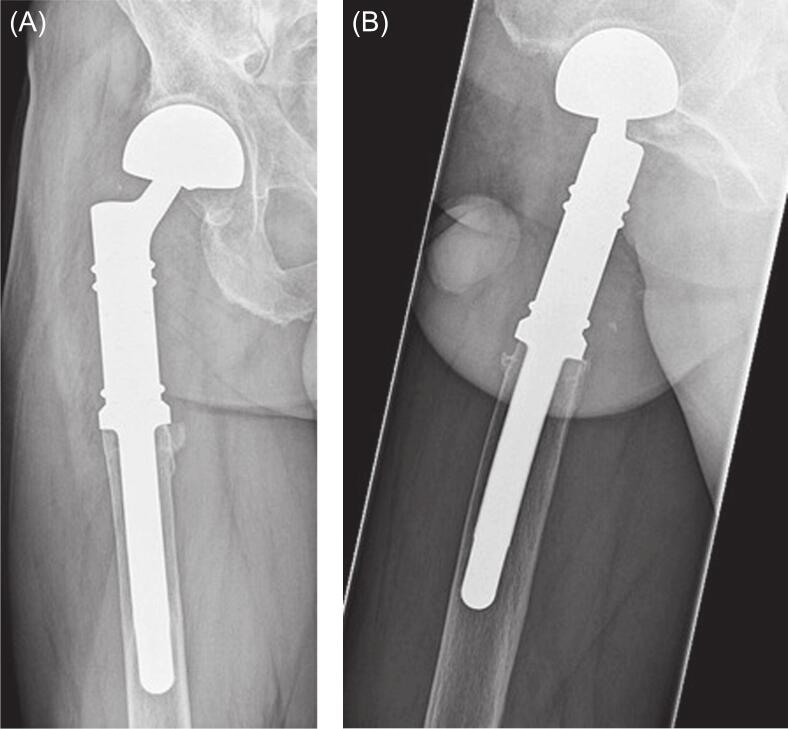
Conventional anteroposterior (1 A) and lateral (1B) radiographs, taken four years after resection of 12 cm of the proximal femur for a grade 2 chondrosarcoma. The defect was reconstructed with an uncemented hydroxyapatite-coated MUTARS® proximal femoral replacement with a bipolar femoral head and an attachment tube. At review, the patient was free from complications and functioned well.

Patients were positioned in the lateral position, and a straight lateral approach was employed. Following tumour resection, the osseous defect was reconstructed with a MUTARS® proximal femoral prosthesis (Fig. 1). Uncemented press-fit fixation was the preferred method of fixation, unless bone quality was deemed insufficient or in case we could not obtain adequate press-fit fixation. Acetabular quality was assessed intra-operatively. When considered sufficient, we preferred hemiarthroplasty with use of a bipolar femoral head [13]. Attachment tubes were fixed to the prosthesis with braided Cerclage (Dall Miles, Kalamazoo, Michigan) to re-attach soft-tissues (*n* = 58, 91%) in an attempt to restore hip function and to reduce the risk of dislocation [14].

Fifty-four reconstructions (84%) were bipolar hemiarthroplasties (Table 1). Three cemented and six uncemented acetabular cups were implanted (*n* = 9, 14%). Fifty stems (78%) were uncemented, of which 34 hydroxyapatite-coated (68%). Twenty-eight (44%) were silver-coated. Median reconstruction length was 18 cm (8 to 45).

All patients received prophylactic cephalosporin antibiotics, and these were usually continued for one to five days after surgery. Median postoperative hospital stay was 8 days (3 to 22). Full weight-bearing was allowed directly after surgery. Follow-up routinely included functional and radiographic examination with conventional radiographs. If a recurrence was suspected, additional magnetic resonance imaging was performed.

### 2.1 Statistical analysis

Summary tables for categorical variables included N and proportion; continuous variables were reported as mean and standard error, or median and range depending on the distribution (Table 1). Complications were recorded and classified according to the failure mode classification as reported by Henderson [15]. Failure was defined as any structural modification of the reconstruction. Repositions after dislocation, debridement with implant retention and isolated liner revision for wear were not considered an implant failure. A competing risks model was employed to estimate the cumulative incidence of implant failure for mechanical failure and infection with patient mortality as a competing event [16]. Risk factors for reconstruction failure and complication rates were assessed using the cause specific hazard model. Cause specific hazard ratio (HR_CS_) and 95% confidence interval are reported. For all tests, a *p*-value of <0.05 was considered significant. Statistical analysis was performed using SPSS 29.0 (IBM Corp, Armonk, NY, USA). Competing risk analysis was performed using package ‘cmprsk’ in R version 4.5.1. [17].

## 3 Results

### 3.1 Mechanical complications

**Fig. 2:**
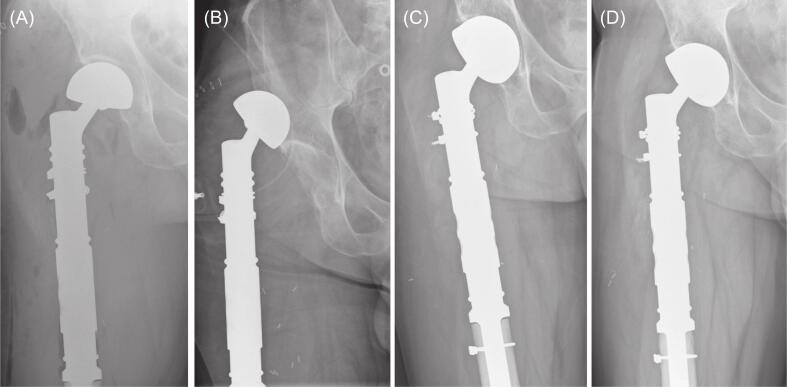
2 A: First postoperative radiograph after proximal femoral reconstruction with a MUTARS prosthesis in a 63-year old male patient with chondrosarcoma. 2B: Dislocation of the bipolar head nineteen days after surgery. 2C: Revision of the bipolar head to a dual-mobility cup. 2D: Two years after conversion to a dual-mobility cup, no complications reported.

Eight dislocations occurred (13%) after a median of seven months (19 days to 5.7 years) (Table 3). Dislocation occurred in three reconstructions with an acetabular cup (3/9, 33%) and in five bipolar hemiarthroplasties (5/54, 9%) (HR_cs_ 5.8, 95% CI 1.4 to 24.8) (Table 4). For the bipolar hemiarthroplasties, all five dislocations occurred in reconstructions longer than 14 cm. Of the dislocated bipolar hemiarthroplasties, one was managed with open reduction and four with implantation of a dual-mobility cup (Fig. 2). Dislocations in total hip arthroplasties were managed with open reduction in two cases and closed reduction in one. Three patients experienced recurrent dislocations, and in all three cases the endoprostheses were eventually removed due to prosthetic infection.

**Table 3:** Complications and limb salvage at final follow-up.

	BHA (%)	THA (%)	Total (%)
**Complications, according to Henderson.**			
Type 1 (soft-tissue, instability)	8 (15)	3 (33)	11 (17)
Type 2 (prosthetic loosening)	0 (0)	0 (0)	0 (0)
Type 3 (hardware failure)	3 (6)	0 (0)	3 (5)
Type 4 (infection)	7 (13)	1 (11)	8 (13)
Type 5 (tumour progression)	10 (19)	1 (11)	11 (17)
Limb salvage achieved at final follow-up	50 (93)	9 (100)	60 (94)

Abbreviations: BHA, bipolar hemiarthroplasty; THA, total hip arthroplasty.

**Table 4:** Univariable cause-specific Cox regression analysis for risk factors associated with dislocation and infection.

Outcome	Variable	HR	95% CI	p-value
Dislocation	Age (per year)	1.01	0.97 to 1.05	0.63
	Male	2.38	0.48 to 11.82	0.29
	THA (ref. BHA)	5.81	1.36 to 24.85	0.02
	Previous surgery	1.26	0.25 to 6.24	0.78
	Resection length ≥ 18 cm	1.09	0.26 to 4.55	0.91
Infection	Age (per year)	0.99	0.96 to 1.03	0.64
	Male	0.74	0.19 to 2.97	0.67
	THA (ref. BHA)	1.03	0.13 to 8.45	0.98
	Duration of surgery (per minute)	1.01	1.00 to 1.02	0.01
	Resection length ≥ 18 cm	4.38	0.53 to 36.39	0.17
	Silver coating	1.41	0.35 to 5.67	0.63

Abbreviations: THA, total hip arthroplasty; BHA, bipolar hemiarthroplasty.

### 3.2 Aseptic loosening (Henderson type 2) was not observed in this cohort

**Fig. 3:**
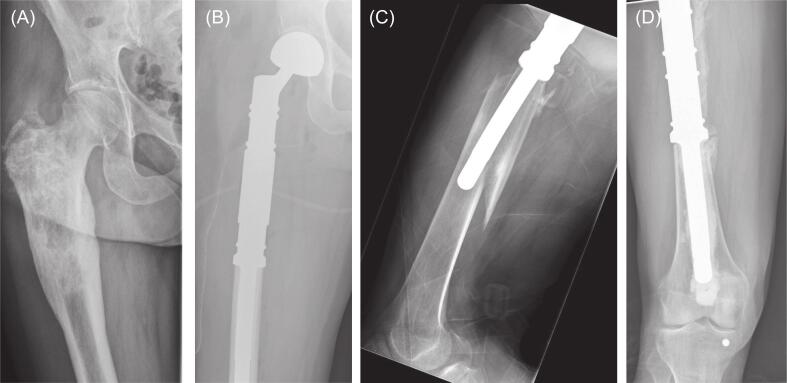
3 A: Preoperative radiograph of a chondrosarcoma of the proximal femur in a 60-year old female patient. 3B: Postoperative radiograph one day after surgery, no complications reported. 3C: Periprosthetic fracture of the femur diaphysis ten years after surgery. 3D: Three months after revision of reconstruction with a cemented stem, resection of the fracture, and extension of MUTARS prosthesis.

Structural failure (Henderson type 3) was observed in three patients (5%). In one patient a periprosthetic fracture occurred 10 years after surgery, which was managed with revision to a cemented stem (Fig. 3). One patient was converted to a total hip arthroplasty 15 years after primary surgery due to radiological evidence of acetabular erosion. Groin pain was only reported by one patient (2%) without radiographic signs of acetabular erosion three years after primary surgery, for which a conversion to a total hip was performed. In twelve reconstructions (16%), a periprosthetic fissure fracture occurred during implantation of the stem and was managed with cerclage without further clinical implications.

### 3.3 Non-mechanical complications

Deep infection (Henderson type 4) occurred in eight patients (13%) after a median of 33 days after the latest surgical procedure (range 12 days to 1.4 years). Five of them were primary infections, four of which were successfully treated with debridement and systemic antibiotics, and one with one-stage revision surgery. Three patients had an infection after a secondary procedure (three open reductions for dislocation) and needed a two-stage revision, two of which were successful. The other failure necessitated exarticulation through the hip due to persistent chronic infection. The duration of surgery differed between infected (mean 4.7 h, range 2.1 to 8.0) and non-infected (3.6 h, 1.6 to 9.0) primary reconstructions (HR_cs_ 1.0, 95% CI 1.0 to 1.0) (Table 4). The percentage of infections in primary reconstructions was equal to 6/37 (16.2%) and 1/25, (4.0%) for reconstructions ≥18 cm and reconstructions <18 cm respectively. The cause specific hazard ratio was equal to 4.4 (95% CI 0.5 to 36.4). Of the primary reconstructions, infection occurred in four silver coated prostheses (4/28, 14%) and four non‑silver coated prostheses (4/36, 11%).

### 3.4 Reconstruction status at final follow-up

**Fig. 4:**
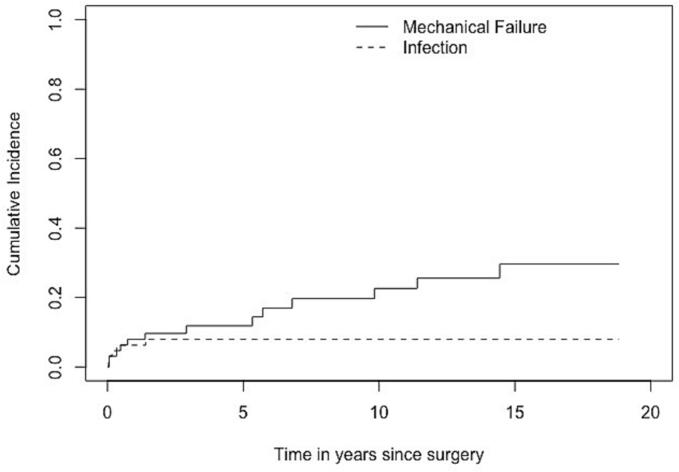
Cumulative incidence of reconstruction failure for mechanical failure and infection.

At final follow up, 60 patients (94%) had a proximal femoral endoprosthesis in situ. Three exarticulations were performed (5%) and one hindquarter amputation (2%). During follow-up, one or more reoperations were undertaken in 27 patients (42%). With failure for mechanical reasons (Henderson type 1–3) as the end-point, the cumulative incidence of reconstruction failure at five, ten, and fifteen years was 11.8% (95% CI 3.4–20.3), 22.6% (95% CI 10.1–35.2), and 29.7% (95% CI 14.4–44.9), respectively. With failure due to infection as the end-point, the cumulative incidence of reconstruction failure at four years reached 8.4% (95% CI 1.2–14.8), and remained stable thereafter (Fig. 4).

## 4 Discussion

Endoprosthetic proximal femoral replacement is a well-established technique in treatment of bony malignancies and has been widely reported in the literature [6], [18], [19], [20]. However, there is a lack of long-term follow up data focussing on patients with longer expected survival, especially patients with non-metastatic disease. Furthermore, clinical outcome specifically of the MUTARS® system is scarce. In the current study, we assessed complications, risk factors for complications, and cumulative incidences of implant failure of proximal femoral replacement with MUTARS® modular endoprostheses for primary tumours.

Dislocation occurred in 9% (5/54) of our bipolar hemiarthroplasties and in 33% (3/9) of the total hip arthroplasties. Previously, Gosheger reported on sixteen patients with reconstruction of the proximal femur using a MUTARS® proximal femur and a bipolar head [21]. None of the bipolar reconstructions that were reinforced with an attachment tube dislocated. The rate of dislocation in our bipolar hemiarthroplasties was comparable to the rate reported by most previous authors on bipolar hemiarthroplasties (5–7%) [1], [7], [22], [23], [24], although others reported a higher rate of dislocation (20%) [25]. Several authors have reported that bipolar femoral heads are associated with a lower risk of dislocation as compared to unipolar heads [1], [7], [13]. Chandrasekar, on the other hand, reported a dislocation rate of only 3% in 91 patients with a unipolar hemiarthroplasty [26]. They, however, started using bipolar femoral heads due to a high risk (5%) of acetabular wear after short term follow up (25 months, 0 to 60). In the current study, one bipolar hemiarthroplasty was converted to a total hip arthroplasty due to radiographic evidence of acetabular erosion. Additionally, one patient underwent conversion to total hip arthroplasty because of persistent groin pain, despite the absence of radiographic signs of acetabular erosion. It thus appears that the use of a bipolar femoral head does not eliminate the risk of acetabular erosion. Drexler previously reported on the radiographic outcome of 65 patients that underwent bipolar arthroplasty for a proximal femoral tumour. Three of their patients (4.6%) developed pain, protrusion and degenerative changes for which a surgical intervention was indicated, all within 5 years follow-up [27]. Bernthal reported a conversion rate to total hip arthroplasties of 5.8% after a mean follow-up of five years [28]. The same study group reported a comparable conversion rate (6%) in an expanded cohort with a mean follow-up of 7 years [24]. Both concluded that routine arthroplasty of the acetabulum was not justified because of the limited number of re-interventions for groin pain and protrusion. We agree with their conclusion for several reasons. First, failure of hip arthroplasty is more common in young, active, high-demand patients [29]. In these patients, loosening or wear of the acetabular component is the leading cause for revision [13], [30], making it reasonable to avoid acetabular resurfacing when possible. Second, bipolar femoral heads appear to reduce the risk of dislocation while maintaining an acceptable risk of acetabular erosion. In our study, even after long-term follow-up, the revision rate due to acetabular erosion was low. Von Roth evaluated revision due to acetabular erosion in conventional bipolar hemiarthroplasty with a minimum follow-up of 20 years and reported a cumulative incidence of 1.4% [31]. Based on these findings, we recommend hemiarthroplasty with use of a bipolar head.

We did not observe any cases of loosening in our cohort. Aseptic loosening has been reported to be among the leading causes for failure in several large series on endoprosthetic reconstruction for bone tumours [10], [21], [32]. It appears that loosening is particularly of concern in reconstructions around the knee [21], [33]. In the current series, 53% of the reconstructions were uncemented hydroxyapatite-coated. Hydroxyapatite coating enhances bony ingrowth and is therefore supposed to guarantee long-term stable fixation [34]. We currently consider uncemented hydroxyapatite-coated implants the most viable option for durable fixation of endoprostheses in patients with good life expectancy [33]. In case of poor bone quality or an altered anatomic shape, cemented fixation is preferred.

Deep infection occurred in 13%, and 6% failed as a result. Because of the good possibilities of soft-tissue coverage, one may expect a limited risk of infection after reconstruction of the proximal femur. However, the risk of infection remains high, plausibly due to extensive soft-tissue resections, implantation of massive prostheses, adjuvant therapies and a long duration of surgery. In a large study on infection in orthopaedic oncology, Gradl reported an infection rate of 8% in resections of the hip or proximal part of the femur [35]. Others reported comparable rates of infection (4–13%) [2], [3], [6], [7]. Sanders described a higher infection rate of 20% in lower-extremity reconstructions, although their longer follow-up likely resulted in a higher observed lifetime risk of infection, mainly due to the continuous need for revisions for mechanical reasons and subsequent risk of infection [36]. Our series show that primary infection can be adequately managed with surgical debridement. We were unable to demonstrate the efficacy of silver coatings. Wafa and Hardes reported an added value for silver coatings for both primary and revision surgery [37], [38]. Therefore, we will continue with routine employment of silver coated implants. We observed that prolonged surgical duration was associated with a higher risk of infection, consistent with previous findings in primary joint arthroplasty [39]. If attachment tubes contribute to the risk of infection remains controversial.

At the final follow-up, limb salvage was achieved in 94% of patients. The primary reconstruction could be retained in 84% of cases. Nevertheless, the proportion of patients requiring at least one reoperation was considerable (42%).

Our study has several limitations. This is a retrospective cohort study with its inherent shortcomings. Due to limited sample size, multivariate analyses could not be performed. Incomplete data on patient- and physician-reported outcomes impeded evaluation of functional outcome. In addition, the exact mechanism of dislocation was not consistently documented and precluded reliable analysis. Because of small patient numbers, we were unable to elucidate important controversies in proximal femoral reconstruction such as the use of attachment tubes and silver coatings. Nevertheless, this study provides value through its long-term follow-up (>10 years). To the best of our knowledge, this is the longest follow-up reported for MUTARS® Proximal Femoral Replacement to date. Furthermore, the cohort is relatively homogenous, consisting exclusively of patients who received a MUTARS® proximal femoral replacement for a primary tumour.

In conclusion, we were able to show that endoprosthetic replacement of the proximal femur with MUTARS® proximal femoral prostheses with bipolar hemiarthroplasty is associated with an acceptable rate of mechanical complications after long-term follow up. Dislocation is a common complication. The rate of other mechanical complications was very low. Although the infection rate is substantial, half of the infected implants could be retained, and most failed implants were salvaged with a second reconstruction. In patients with primary bone tumours, we recommend the MUTARS® proximal femoral prostheses with bipolar hemiarthroplasty.

## CRediT authorship contribution statement

**P.T.J. Sanders:** Writing – review & editing, Writing – original draft, Visualization, Resources, Methodology, Formal analysis, Data curation. **S.F. van de Vusse:** Writing – review & editing, Writing – original draft, Visualization, Software, Resources, Methodology, Formal analysis, Data curation. **M.P.A. Bus:** Writing – review & editing, Supervision, Methodology, Conceptualization. **M. Fiocco:** Writing – review & editing, Software, Methodology, Formal analysis. **J.A.M. Bramer:** Writing – review & editing, Resources. **G.R. Schaap:** Writing – review & editing, Resources. **M.A.J. van de Sande:** Writing – review & editing, Supervision, Resources, Project administration, Conceptualization. **P.D.S. Dijkstra:** Writing – review & editing, Supervision, Resources, Project administration, Conceptualization.

## Declaration of competing interest

The authors declare the following financial interests/personal relationships which may be considered as potential competing interests: M.A.J. van de Sande reports financial support was provided by Implantcast GmbH. P.D.S. Dijkstra reports financial support was provided by Implantcast GmbH. M.A.J. van de Sande reports a relationship with European Musculoskeletal Oncology Society that includes: board membership. If there are other authors, they declare that they have no known competing financial interests or personal relationships that could have appeared to influence the work reported in this paper.

**Table:** 

Author	Year	Number of patients	Indications	Follow-up	Type of implant	Stem fixation	Articulation type	Dislocation	Loosening	Infection	Implant survival
Ashford [41]	2010	63	Mets	Survivors: mean 30 months (10 to 48) Deceased: mean 21 months (1 to 85)	Custom-made or METS modular (Stanmore)	Cemented (HA-collar used with the METS in the majority of cases)	THA 84% Bipolar hemi 13% MoM resurfacing 3%	35%	N/R	6%	N/R
Bernthal [28]	2010	86	Primary 78% Mets 22%	Mean 64.4 months (3–291 months)	HMRS (Howmedica)	Cemented	Bipolar hemi	3%	3%	1%	97% at five years 84% at ten years 56% at twenty years
Trikha [24]	2023	122	Primary 78% Mets 22%	Mean 7 years (± 8 years)	Zimmer Biomet, Stryker, DePuy Synthes, Howmedica	Cemented	Bipolar hemi	5%	3%	3%	97% at five years 81% at ten years 69% at twenty years 51% at thirty years
Bickels [42]	2000	57	Primary Mets 11%	Mean 80 months (24–418)	Kotz (Howmedica)	Cemented	Bipolar hemi 86% Unipolar hemi 14%	2%	5%	4%	N/R
Chandrasekar [6]	2009	100	Mets 65% Primary 25% Other 10%	Mean 25 months (0 to 60)	METS modular (Stanmore)	Cemented	Unipolar hemi 91% THA 9%	6%	3% (of the patients assessed at final follow-up)	7%	95% at one year 91% at five years
Donati [43]	2001	25	Primary 100%	Mean 12 years (10 to 16.5)	KMFTR (Howmedica)	Uncemented	Bipolar hemi	2%	10%	2%	N/R
Drexler [27]	2015	65	Primary 76% Mets 23%	Mean 9 years (2 to 11)	HMRS (Howmedica) MUTARS (Implantcast)	N/R	Bipolar hemi	3%	1%	0%	88% at 17 years
Farid [1]	2006	52	Primary 63% Mets 35%	Primary: median 50 months (24 to 326) Mets: median 45 (29 to 144)	N/R	Cemented	Bipolar hemi 77% THA 23%	6%	10%	4%	86% at ten years
Finstein [23]	2007	62	Mets 52% Primary 48%	Mean 5 years (0.1 to 21.6)	One-piece PFRP (Biomet) 16% GMRS (Stryker) 84%	Cemented	Bipolar hemi	5%	10%	5%	89% at two years 86% at five years 62% at ten years (revision-free survival)
Gosheger [21]	2006	41	Primary		Implantcast	Uncemented 32% Cemented 68%	Bipolar hemi 57% THA 43%	7%	N/R	20%	79% at five years
Ilyas [25]	2002	15	Primary	Mean 6.7 years (3 to 10)	HMRS (Howmedica)	Uncemented	Bipolar hemi	20%	7%	13%	N/R
Kabukcuoglu [44]	1999	54	Primary	Mean 9 years (5 to 24)	Custom-made (Stanmore)	Cemented	THA	11%	11%	6%	77% at ten years 57% at 20 years
Menendez [7]	2006	96	Mets 75% Primary 25%	Mean 18 months (1 to 129)	HMRS (Howmedica)	Cemented	Bipolar hemi 65% THA 35%	10%	N/R	6%	94% at two years 82% at five and ten years
Natarajan [5]	2003	44	Primary 89% Mets 11%	Mean 57 months (24 to 144)	Custom-made (manufacturer N/R)	N/R	THA 61% Hemi 39%	7%	N/R	7%	97% at five years (mechanical failure as end-point)
Potter [22]	2008	61	Mets 64% Primary 36%	Survivors: mean 55 months (24 to 152) Deceased: mean 12 months (2 to 34)	GMRS (Stryker)	Cemented	Bipolar hemi	7%	3%	5%	79% at five years
Pala [8]	2021	35	Primary Mets	Mean 3.4 years (0.1–16)	MUTARS (implantcast)	N/R	N/R	9%	0%	0%	82% at five years 63% at ten years
Tsukushi [45]	2022	49	Mets 63% Primary 37%	Mean 35 months (24–53)	KMLS (JMOG)	Uncemented	N/R	2%	2%	2%	88% at two years 80% at four years
Current study	2026	64	Primary	Median 10.1 years	MUTARS (implantcast)	Uncemented 78% (HA-coated 68%) Cemented 22%	Bipolar hemi 84% THA 15%	13%	0%	13%	88% at five years 77% at ten years 70% at fifteen years

Bipolar hemi = bipolar hemiarthroplasty.

MoM resurf = metal-on-metal hip resurfacing.

Unipolar hemi = unipolar hemiarthroplasty.

Primary = primary tumour.

Mets = metastatic disease.

N/R = not reported.

THA = total hip arthroplasty.
